# EDUCATION AS A MODERATOR IN THE ASSOCIATION OF SLEEP DURATION AND DEPRESSIVE SYMPTOMS

**DOI:** 10.1093/geroni/igae098.3519

**Published:** 2024-12-31

**Authors:** Wuyi Dong, Yan-Jhu Su, ChangPu Liang, Dongfang Hong

**Affiliations:** University of Massachusetts Boston, Boston, Massachusetts, United States; University of Massachusetts Boston, Boston, Massachusetts, United States; University of Massachusetts Boston, Boston, Massachusetts, United States; University of Massachusetts Boston, Boston, Massachusetts, United States

## Abstract

Optimal sleep duration is critical for older adults’ health and either too long or too short sleep duration potentially leads to mental disorders such as depression, anxiety, and distress. This study explores the association between sleep duration and depression among older adults, with a focus on whether education achievement buffers the association. To create a pooled cross-sectional dataset, we combined data from the National Health and Nutrition Examination Survey (NHANES) of two consecutive waves (2015-16 and 2017-18). Our analysis includes a weighted sample of individuals aged 60 and older without missing responses (n=9,254). We measured sleep duration for three categories based on weekday reports: short sleep (< 7 hours), optimal sleep (7 to 9 hours), and long sleep (>=9 hours). Depression was evaluated using total scores from a 9-item Patient Health Questionnaire (PHQ; range: 0-27). Through multiple linear regression models—adjusting for demographics characteristics, self-reported health, health behaviors, and living arrangements, we found either people with short sleep duration or long sleep duration is associated with higher depression symptoms (β=1.28, p<.001 and β=1.24, p<.001, respectively), compared to optimal sleep duration individuals. The interaction effect showed that higher education levels (college or above) significantly decrease depression associated with longer sleep (β=-1.66, p<.05). These findings suggest that both insufficient sleep and excessive sleep increase the depression risks among older adults, while higher education may have protective effects against mental health challenges related to non-optimal sleep durations.

